# Relationship between Health Insurance Status and the Pattern of Traditional Medicine Utilisation in Ghana

**DOI:** 10.1155/2015/717926

**Published:** 2015-08-11

**Authors:** Razak Mohammed Gyasi

**Affiliations:** Complementary and Alternative Therapy Research Unit, Department of Geography and Rural Development, PMB, Kwame Nkrumah University of Science and Technology, Kumasi, Ghana

## Abstract

This paper examines the relationship between national health insurance status and the pattern of traditional medicine (TRM) use among the general population in Ghana. A retrospective cross-sectional survey of randomly sampled adults, aged ≥18 years (*N* = 324), was conducted. The results indicate that TRM use was high with prevalence of over 86%. The study found no statistically significant association between national health insurance status and TRM utilisation (*P* > 0.05). Paradoxically, major sources of TRM, frequency of TRM use, comedical administration, and disclosure of TRM use to health care professionals differed significantly between the insured and uninsured subgroups (*P* < 0.001). Whereas effectiveness of TRM predicted its use for both insured [odds ratio (OR) = 4.374 (confidence interval (CI): 1.753–10.913; *P* = 0.002)] and uninsured [OR = 3.383 CI: 0.869–13.170; *P* = 0.039)], work experience predicted TRM use for the insured [OR = 1.528 (95% CI: 1.309–1.900; *P* = 0.019)]. Cultural specific variables and health philosophies rather than health insurance status may influence health care-seeking behaviour and TRM use. The enrollment of herbal-based therapies on the national health insurance medicine plan is exigent to ensure monitoring and rational use of TRM towards intercultural health care system in Ghana.

## 1. Introduction

The growing interest and uptake of traditional medicine (TRM) in both local and global scales have been recognised and documented by various studies in both high-income and low- and middle-income countries [1–7]. Prescriptively, people in the academia, health professionals, policy-makers, and the general public have expressed concerns regarding the safety, efficacy, quality control, and regulatory subtleties of TRM utilisation. Nevertheless, the Director-General of WHO touted at the International Conference on Traditional Medicine for South-East Asian Countries that “traditional medicines, of proven quality, safety and efficacy, contribute to the goal of ensuring that all people have access to care” [8]. Hitherto, a body of research has reiterated the upsurge demand globally, for herbal medicines, herbal health products, herbal pharmaceuticals, nutraceuticals, food supplements, and herbal cosmetics due to the growing recognition of these natural products as mainly nontoxic and having fewer side effects [9–11]. Gyasi et al. [12] and Peltzer and Mngqundaniso [11] among others have independently justified the safety of herbal medicine due to their naturality and neutrality.

Herbal medicines, traditional treatments, and traditional practitioners are the main source of health care and sometimes the only source of care for most people in economically less developed countries. Indeed, TRM plays a crucial part alongside the conventional medical practice in meeting the health needs of the vast majority of populations in low- and middle-income countries [3, 13]. In Ghana, orthodox and alternative medicines operate side by side in the provision of health care for the citizenry [14, 15]. Among Ghanaians, TRM epitomises health care resource that is close to homes, accessible, affordable, culturally acceptable, and trusted by the majority [8, 16]. In the wake of escalating costs of classic scientific health care, TRM redeems people of poor health, battling with relentless rise of both communicable and chronic noncommunicable diseases [17].

Overwhelming evidence shows that out-of-pocket payment which remains a major means of health care financing across developing countries potentially plummets health care use notably among the resource constrained and underserved [18–25]. Ruinous user fee for health care has the tendency to thrust the entire households into destitution leading to poor health outcomes. This reaffirms the WHO's publication which explicates that out-of-pocket health payment is the least efficient and most inequitable means of financing health care, preventing people from seeking medical care, and may exacerbate poverty [18]. It has been unreservedly reported that individuals without health insurance present poorer health outcomes [26]. This has a strong influence on their health seeking behaviour and diverse medical decisions.

In countries such as China, South Korea, and Vietnam, health insurance fully covers TRM treatment and products [27]. In the Republic of Korea, WHO [8] observed that a national medical insurance programme has covered Korean TRM services since 1987 and currently private insurance also covers TRM services. In Vietnam, TRM practitioners are able to practise in both public and private hospitals and clinics and government insurance fully covers acupuncture, herbal medicines, and TRM treatment [8]. Other countries,* inter alia*, United Kingdom, Japan, Germany, Australia, and the United States, have partial insurance coverage for TRM consumption [28]. Health insurance coverage can lead to a substantial increase in the use of TRM services. Report shows that Americans spend more on complementary and alternative medicine (CAM) than on all hospitalisation [29, 30]. Australians also spend more on CAM than on all prescription drugs due to insurance coverage [31]. Chen et al. [32] found that the frequency of Taiwanese who had visited traditional Chinese medicine (TCM) services within previous one year in 2001 was as high as 28.4% because of the inclusion of TCM in the national health insurance in Taiwan.

Over the past decade, there has been an increased experimentation of the social health insurance policy in a number of African countries, namely, Benin, Nigeria, Rwanda, Kenya, Senegal, Tanzania, and Ghana [33–35]. However, the coverage is limited to the orthodox health care delivery. The public therefore continues to make out-of-pocket payments for TRM services [28]. In Ghana, for example, herbal unit has been established in 17 hospitals and clinics as a driving force for integrative medicine. Notwithstanding, the consultations, treatment, and dispensing of herbal medicine and products in these health facilities are still not covered under the National Health Insurance Package. Barimah [36] argues that as we look for the successful implementation of the National Health Insurance Scheme to fortify robust health services any attempt to include traditional healers and their practices may be the right way forward. Insurance coverage for TRM sector for resource-poor and underserved is a conduit for reducing health care financial burden and for improving health status of the majority.

In a study of predictors of traditional medicines utilisation in the Ghanaian health care practice with Ashanti example, Gyasi et al. [13] observed that low-income earners are more likely to consume TRM than the high-income earners. Using triangulated research approach, Gobah and Zhang [19] noted that health insurance is a key determining factor for health care and treatment seeking and using modern health care facilities. Poverty, therefore, remains a “push” factor in relation to TRM use. Individuals who cannot afford modern health care bills mostly utilise TRM [13]. The question that lingers on the minds of many is whether there has been a change in the pattern of use of TRM when National Health Insurance Scheme is in vogue which apparently does not cover the practices of TRM in Ghana. This will provide the avenue to ascribe the need to support and sustain the growth of traditional medical system. Unfortunately, there is paucity of research in this regard in the Ghanaian context. In an attempt to address this research gap emerges the current study. The study therefore aimed at analysing the relationship between national health insurance status and the pattern of TRM use in the Ashanti Region, Ghana.

## 2. Data and Methods

### 2.1. Study Design and Sampling Procedure

This study was part of a larger research project which investigated the factors influencing traditional medicines utilisation in Ghana with Ashanti example. The study espoused a retrospective cross-sectional and quantitative survey covering rural and urban character. This population-based study involved adults (≥18 years) who could willfully decide for themselves as regards treatment options if need be. The sampling technique was multistage. In the first stage, the entire study region was clustered into rural and urban subdistricts, based on the definition of Ghana Statistical Service (GSS) [14]. Two political and administrative districts, namely, Sekyere South District (designated as rural district) and Kumasi Metropolis (representing the urban districts), were selected in sequence using cluster and simple random sampling. In the second stage, simple random sampling was used in the selection of 5 study settlements from each of the study districts. Akrofonso, Bedomase, Bepoase, Boanim, and Domeabra were selected from the rural Sekyere South District whilst Atonsu, Ayigya, Nhyiaeso, Old Tafo, and Suame were selected from the Kumasi Metropolis.

To ensure representativeness and generalisability of the study findings by whipping down sampling bias, the sample size (*n*) necessary for this study was determined based on a 70.0% estimated prevalence (*p*) of TRM use in Ashanti Region [14, 15, 37] with a 5.00% level of uncertainty or precision (*d*) using the formula *n* = *t* ^2^ *pq*/*d* ^2^ [38–40], where *t* ^2^ denotes a 95.0% confidence (5.00% level of significance) and *q* = 1 − *p*. With this formula, a minimum required sample size of 323 approximately was estimated. In the third stage, a total of 324 eligible participants were selected from the study prefecture. This stage was divided into two tiers. First, systematic random sampling was applied to select the houses or compounds. Then, eligible respondents were obtained from the household units through simple random sampling procedure.

### 2.2. Data Collection

Formal face-to-face household-level interviewer-administered questionnaire was used in the primary data collection. The interviews were done by trained research assistants from the Medical and Health Geography Class at the Department of Geography and Rural Development, KNUST, Kumasi. Whilst the researcher closely monitored data collection processes during field work, spot-checks and rechecks on completed questionnaire were executed to ensure quality control. The questionnaire was translated into Asante Twi (the predominant dialect in the study prefecture) and translated back into English to ensure content validity and reliability of the instrument. Participation in the study was entirely voluntary, and an informed consent was therefore obtained from each respondent who agreed to partake in the study. Moreover, the study protocol was obtained from the Committee on Human Research Publication and Ethnics (CHRPE), School of Medical Sciences at Kwame Nkrumah University of Science and Technology (KNUST), and Komfo Anokye Teaching Hospital (KATH), Kumasi (CHRPE/AP/260/14), following the principles resonated by the Declaration of Helsinki [41, 42].

### 2.3. Variables and Preferred Cutoff Values

The outcome variable was traditional medicine utilisation, operationalised as nonuse or use of TRM over the last one year preceding the survey. In this study, TRM was operationalised as medical products and practices that are not part of standard care such as medical doctors, doctors of osteopathy, and allied health professionals (nurses and physical therapists) practice. The outcome variable was assessed by self-reporting through an answer to the question: “Have you ever used any form of TRM or accessed the services of traditional medical practitioners (TMPs) for your medical or spiritual and psychological problem within the last 12 months?” The response was entered as a dichotomous variable and coded as 1 = yes or 0 = no. The insurance status was defined as insured = 1; uninsured = 2. In this case, the insured consisted of individuals who had unexpired national health insurance card and therefore could, to some extent, access free medical care within the past 12 months preceding the survey. Other exposure variables were categorised into demographic (age, sex, religious background, marital status, household size, and ethnicity), socioeconomic (educational level, household income level, health insurance status, employment status, residency, nature of occupation, and work experience), and biopsychosocial and anthropological variables (perceived efficacy, nature of disease, safety or side effects, and quality of TRM).

The exposure variables were further categorised as age (<20 = 1; 20–29 = 2; 30–39 = 3; 40–49 = 4; 50–59 = 5; ≥60 = 6), sex of respondent (male = 1; female = 2), marital status (married = 1; single = 2), residence (urban = 1; rural = 2), education (never been to school = 1; basic = 2; secondary = 3; tertiary = 4), income status (≤GH¢100 = 1; GH¢101–GH¢300 = 2; GH¢301–GH¢500 = 3; GH¢501–GH¢1000 = 4; ≥GH¢1001 = 5), ethnicity (Akan = 1; Ewe = 2; Ga-Dangme = 3; Mole-Dagbani = 4; Guan = 5; Gurma = 6), religious denomination (Christianity = 1; Islam = 2; Traditional African Religion = 3; others = 4), household size (≤3 = 1; 4–6 = 2; 7–10 = 3; 11–15 = 4; 16–19 = 5; ≥20 = 6), employment status (employed = 1; unemployed = 2), nature of occupation (farming = 1; artisanal work = 2; civil/public service = 3), presence of chronic noncommunicable diseases (yes = 1; no = 2; do not know = 3), perceived safety of TRM (poor = 1; satisfactory = 2; good = 3; very good = 4), and perceived efficacy of TRM (poor = 1; satisfactory = 2; good = 3; very good = 4).

### 2.4. Data Management and Analysis

Descriptive statistics were conducted to describe the background characteristics of the study sample. A multiple logistic regression analysis (backward stepwise method) was conducted separately to identify the factors associated with TRM use for the insured and the uninsured respondents. The odds ratio (OR) and a 95% confidence interval (CI) were determined for each explanatory variable. Bivariate analyses with Pearson's Chi-square test and Fisher's exact test were used to identify the associations between variables and to compare health insurance status of the respondents and use of TRM. All analyses were done through the Predictive Analytics Software (PASW) for Windows application programme (version 17.0) with probability value of *P* < 0.05 as statistically significant. Data were organised and presented by frequency tables and proportionate counts.

## 3. Results

### 3.1. Sample Characteristics

The background characteristics of the study participants by health insurance status are presented in Table 1. The total sample included 324 adults, ≥18 years from rural and urban communities in Ghana. The majority (71.6%) of the sample reported to have enrolled in the National Health Insurance Scheme, hence insured. There were more female (59.9%) than male (40.1%) participants. In all, most respondents (52.5%) were younger, within the age cohort of ≤39 years, married (62.0%), schooled up to the basic level (63.9%), employed (86.5%) but in the informal sector (69.4%) including petty trading, farming, and artisanal economic ventures. This low level of educational attainment and its corresponding informal nature of occupation the respondents engaged in actually mirrored the low-income levels among the study sample with the majority (70.6%) receiving ≤GH¢300.00 (US$100.00) per month.

Approximately, 78.0% of the participants were from the Akan ethnic group. Similarly, about 82.0% professed Christian faith. In a bivariate analysis to compare the baseline sample characteristics between the insured and uninsured participants, the study found statistically significant differences between the two subgroups in relation to sex (df = 1; *N* = 324, *P* = 0.002), marital status (df = 1; *N* = 324, *P* = 0.032), nature of occupation (df = 5, *N* = 324, *P* = 0.021), and household size (df = 1; *N* = 324, *P* = 0.013). Other variables did not differ significantly between the insured and uninsured study participants (*P* > 0.05) (see Table 1).

### 3.2. Relationship between Health Insurance Status and Pattern of TRM Utilisation

Overall, more than 86% of the sample reported use of various modalities of TRM for different ailments within the 12-month period preceding the survey. A slight difference in TRM use between the insured and uninsured was observed in nominal terms but this difference was not statistically significant (85.3% versus 88.0%; *P* > 0.05) based on Pearson's Chi-square fitness-of-test performed (see Table 2).

Table 3 shows the relationship between health insurance status of the respondents against sources of TRM, frequency of TRM use, co-TRM use with orthodox medicine, disclosure of TRM to health care professionals, and endorsement of full integration of TRM into the mainstream national health delivery system. Regarding the TRM sources, there was a statistical significant association between various sources of TRM and insurance status. Whereas the insured were more likely to obtain TRM from consultations with TMPs (25.0% versus 13.0%, *P* < 0.001), the uninsured were more likely than the insured to access TRM by self-administration (54.3% versus 45.3%, *P* < 0.001) and purchases from pharmacy shops (23.9% versus 20.7%, *P* < 0.05). On the contrary, the uninsured were more likely to have used TRM once within the period of investigation (*χ* ^2^ [1, *N* = 58] = 3.379, *P* < 0.05).

Participants enrolled in the National Health Insurance Scheme had the likelihood to concomitantly utilise TRM with prescribed drugs obtained chiefly from health facilities (df = 1; *N* = 86, *P* < 0.001). However, the study showed no statistically significant association between the health insurance status and disclosure of TRM use to health care professionals (*P* > 0.05). The insured and the uninsured alike showed less importance in divulging to health care providers as regards the consumption of various forms of TRM. Similarly, the study found no significant difference between the insured and uninsured respondents in relation to the endorsement of integrating TRM into the national health care delivery system (*P* > 0.05).

Stratifying by health insurance status, Table 4 depicts the analysis of the multiple logistic regression for the insured and uninsured respondents. For the insured, those who had more work experience were nearly twice as likely to use TRM as those who had less work experience [OR = 1.528 (95% CI: 1.309–1.900; *P* = 0.019)] and those who perceived TRM to be effective were more than four times more likely to report TRM use [OR = 4.374 (CI: 1.753–10.913; *P* = 0.002)]. For the uninsured, compared to those who perceived TRM to be less effective, those who considered TRM to be more effective were more likely to use TRM [OR = 3.383 (CI: 0.869–13.170; *P* = 0.039)].

## 4. Discussion

This is the first current survey investigating the relationship between health insurance status and use of TRM in a Ghanaian rural and urban context. The study found among the general adult population in rural Sekyere South District and Kumasi Metropolis of Ashanti Region a high prevalence of TRM use. TRM utilisation rate of over 86% is comparable to findings of other studies in low- and middle-income countries [43–47]. The study found effectiveness of TRM to predict TRM use by the insured and uninsured subgroups. The perceived efficacy of TRM use is ubiquitous and apparently cuts across whether or not being enrolled in the National Health Insurance Scheme. People that perceived TRM as effective in managing and preventing various ailments had the highest odds and therefore were more likely to report TRM use among both the insured and the uninsured. This finding is consistent with other previous research outputs that have reported vehemently that a growing utilisation of various modalities of TRM is subject to their efficacy and potency [1, 12, 16, 29, 31, 45, 46].

The study revealed no statistically significant relationship between TRM use and health insurance position. It is not surprising to find no differences in the utilisation of TRM regardless of the status of health insurance in a society where traditional medical therapies have been in the culture. This relationship defies previous studies that found health insurance status of the study sample to influence TRM usage [28–30, 32]. In Ghana, like other sub-Saharan African countries, national and mutual health insurance covers only the prescribed drugs and medical consultations with the orthodox health care professional, having no consideration for traditional medical care [13, 18–21]. Yet, TRM use remains universal among the insured and the uninsured in Ghana. This is subject to the fact that TRM evolved and developed from different philosophical grounds than the modern conventional medicine. Generally, the two medical systems have a divergent approach in diagnosis, disease management, and prevention. Patients employ TRM and allopathic medicine for distinctly different needs [48]. Each of the medical systems is effective in dealing with different kinds of ailments. TRM could treat or cure certain diseases that are not likely to be treated by orthodox medicines and the reverse is true. For example, in the public perceptions of the role of traditional medicine in the health care delivery system in Ghana, Gyasi et al. [16] noted that TRM was effective in treating fracture or broken bone, diseases of psychic nature, and other “tropical diseases” as reported by study respondents. The nature of diseases and its associated anguish may be instrumental in health care use compared to health insurance status [49]. Furthermore, health-seeking behaviour of individuals has a connotation with people's cultural beliefs, tradition, and religious denomination. People might therefore continue relying on TRM for certain disease whether or not they are enrolled in the National Health Insurance Scheme. Cultural attitudes and ethnic group controls explain universality in TRM use, even among those who are insured [50].

Moreover, most people perceive certain aspects of TRM with particular reference to the biologically based therapies as natural, less toxic, with little or no side effects as compared to the prescribed drugs [51]. In this regard, health insurance status of the respondents has limited associative effect on the utilisation of TRM. Ready availability that informs easy acquisition of traditional medical products (sometimes from farms and neighbourhoods or backyard gardens) and the opportunity to purchase these readily available products from open markets, mobile peddlers, herbal shops, and pharmacy shops cannot be glossed over in explaining the difference in TRM utilisation between the insured and the uninsured. The easy access to TRM incessantly pulls respondents whether insured or uninsured to utilise TRM without question. This partly accounts for the widespread use of TRM by the study participants irrespective of their health insurance position.

In a bivariate analysis, the study found statistically significant differences between the insured and uninsured subgroups in relation to sex, marital status, nature of occupation, and household size. Females were more likely to be enrolled in the National Health Insurance Scheme than males (65.1% versus 34.9%). In similar sense, the married had greater propensity to be insured compared with the unmarried (65.1% versus 34.9%). Studies have reported that females have a high demand for health care due to the fragility and relapsing reproductive functions and other health-related challenges of females [49, 52, 53]. As a means to reducing the burden of escalating health care expenditure, more females than males enroll in the national health insurance. The influence of marriage couples and partners is reflected in the difference between insurance coverage between the married and the unmarried respondents.

The uninsured offer full cost recovery for all medications and consultations with a health care provider [36, 54]. This serves as a “push” mechanism for considering other alternative sources for health care. The study provides evidence to suggest that the uninsured access TRM mostly through purchases from pharmacy and/or chemical shops and therefore resort to self-administration and medication with its concomitant challenges. Self-medication without prescription might present serious challenges for the health and quality of life of individual patient. Similarly, the study found a significant relationship between the frequency of TRM use and insurance status. People who are covered under the health insurance have the likelihood to concomitantly utilise TRM with prescribed drugs amidst its positive and negative effects. This depicts the high demand and preference for and the central role TRM plays in maintaining and restoring good health for all manners of people. This has implications for the development and sustainability of TRM in the health care delivery system, particularly in the low- and middle-income countries.

The major strength of this study is that it remains the first population-based study to offer elucidation as regards the specific association between the health insurance status and the pattern of TRM utilisation in the Ashanti Region of Ghana. However, the study is beset with some limitations. Although rural-urban character vis-à-vis gender dimensions was infused, the survey was limited to only two political and administrative districts of the Ashanti Region. Tackling this pitfall was the fact that a representative sample and randomisation procedures were followed in the generation of the study results. The use of cross-sectional survey in retrospect means that recall bias is virtually inevitable. Again, the cross-sectional design could not proffer argument about whether or not the use of TRM is increasing over time. The selection bias was unavoidable. This is reflected in the oversampling of the insured and female participants. This could result in either underreporting or overreporting of TRM use thereby painting a blurry picture from the reality. It therefore comes with a difficulty to ascribe a vivid causal relationship between the study variables of interest. Replication of this research and follow-up studies in other regions would be useful to confirm the consistency of the findings of the current research.

## 5. Conclusion

Traditional therapy use continues to grow in the global landscape in general and in the Ashanti Region of Ghana in particular where the study found over 86% of the participants using one form of TRM or another. The study has shown that there is no difference between the insured and the uninsured participants as regards TRM use although major sources of TRM, frequency, and disclosure of TRM use to health care providers differ significantly between the subgroups. TRM use may be influenced by culture, personal philosophies, and belief systems of people [1]. Given the wholesale consumption of TRM, all interventions geared towards improving and sustaining the quality of TRM should be marshaled by the government via the national health care-related institutions and concerned philanthropists. Again, the dispensing and administration of herbal-based therapies (in health facilities) should be enrolled in the National Health Insurance Scheme's drug list. This will help promote monitoring and rational use of TRM so as to fortify the forward march of full medical integration and intercultural health care in Ghana.

## Figures and Tables

**(a):** 

Variable	National health insurance status						*P* value
	Insured		Uninsured		Total		
	*N* = 232	(71.6%)	*N* = 92	(28.4%)	*N* = 324	(%)	
Age (years)							
<20	5	(2.2)	4	(4.3)	9	(2.8)	0.618
20–29	56	(24.1)	28	(30.4)	84	(25.9)	
30–39	59	(25.4)	18	(19.6)	77	(23.8)	
40–49	44	(19.0)	15	(16.3)	59	(18.2)	
50–59	32	(13.8)	14	(15.2)	46	(14.2)	
≥60	36	(15.5)	13	(14.1)	49	(15.1)	
Sex							
Male	81	(34.9)	49	(53.3)	130	(40.1)	0.002^**∗∗**^
Female	151	(65.1)	43	(46.7)	194	(59.9)	
Residential status							
Urban	115	(49.6)	47	(51.1)	162	(50.0)	0.805
Rural	117	(50.4)	45	(48.9)	162	(50.0)	
Marital status							
Single	81	(34.9)	42	(45.7)	123	(38.0)	0.042^**∗**^
Married	151	(65.1)	50	(54.3)	201	(62.0)	
Educational status							
Never been to school	35	(15.1)	18	(19.6)	53	(16.4)	0.281
Basic education	115	(49.6)	39	(42.4)	154	(47.5)	
Secondary	52	(22.4)	27	(29.3)	79	(24.4)	
Tertiary	30	(12.9)	8	(8.7)	38	(11.7)	
Educational status (partner)							
Never been to school	23	(13.3)	12	(20.0)	35	(15.0)	0.585
Basic education	74	(42.8)	26	(43.3)	100	(42.9)	
Secondary	57	(32.9)	16	(26.7)	73	(31.3)	
Tertiary	19	(11.0)	6	(10.0)	25	(10.7)	
Total	173	(100.0)	60	(100.0)	233	(100.0)	
Religious background							
ATR	5	(2.2)	3	(3.3)	8	(2.5)	0.190^a^
Christianity	193	(83.2)	71	(77.2)	264	(81.5)	
Islamic	28	(12.1)	11	(12.0)	39	(12.0)	
Other	6	(2.6)	7	(7.6)	13	(4.0)	
Employment status							
Employed	197	(86.4)	79	(86.8)	276	(86.5)	0.923
Unemployed	31	(13.6)	12	(13.2)	43	(13.5)	
Total	228	(100.0)	91	(100.0)	319	(100.0)	
Nature of occupation							
Trading	91	(39.2)	21	(22.8)	112	(34.6)	0.021^**∗**^
Farming	33	(14.2)	19	(20.7)	52	(16.0)	
Government	34	(14.7)	9	(9.8)	43	(13.3)	
Artisan	41	(17.7)	20	(21.7)	61	(18.8)	
Schooling	7	(3.0)	6	(6.5)	13	(4.0)	
Others	26	(11.2)	17	(18.5)	43	(13.3)	
Working experience (years)							
1–5	67	(32.8)	30	(37.5)	97	(34.2)	0.616
6–10	48	(23.5)	17	(21.3)	65	(22.9)	
11–15	32	(15.7)	16	(20.0)	48	(16.9)	
16–20	24	(11.8)	9	(11.3)	33	(11.6)	
≥21	33	(16.2)	8	(10.0)	41	(14.4)	
Total	204	(100.0)	80	(100.0)	284	(100.0)	
Tribe/ethnicity							
Akan	185	(79.7)	68	(73.9)	253	(78.1)	0.227^a^
Ewe	8	(3.4)	9	(9.8)	17	(5.2)	
Ga-Dangme	14	(6.0)	5	(5.4)	19	(5.9)	
Mole-Dagbani	18	(7.8)	5	(5.4)	23	(7.1)	
Guan	4	(1.7)	3	(3.3)	7	(2.2)	
Gurma	3	(1.3)	2	(2.2)	5	(1.5)	
Household size							
<3	67	(28.9)	33	(35.9)	100	(30.9)	0.063
4–6	108	(46.6)	27	(29.3)	135	(41.7)	
7–10	43	(18.5)	24	(26.1)	67	(20.7)	
11–15	9	(3.9)	3	(3.3)	12	(3.7)	
16–19	1	(0.4)	2	(2.2)	3	(0.9)	
≥20	4	(1.7)	3	(3.3)	7	(2.2)	
Household monthly income (GH¢)							
≤100	51	(31.5)	25	(42.4)	76	(34.4)	0.273^a^
101–300	62	(38.3)	18	(30.5)	80	(36.2)	
301–500	28	(17.3)	12	(20.3)	40	(18.1)	
501–1000	21	(13.0)	4	(6.8)	25	(11.3)	
≥1001	0	(0.0)	0	(0.0)	0	(0.0)	
Total	162	(100.0)	59	(100.0)	221	(100.0)	

^**∗∗**^ *P* < 0.005; ^**∗**^ *P* < 0.05; ^a^result is based on FET.

**(b):** 

Characteristic	National health insurance status						*P* value
	Insured		Uninsured		Total		
	*N* = 232	(71.6%)	*N* = 92	(28.4%)	*N* = 324	(%)	
Current health status							
Poor	13	(5.6)	4	(4.4)	17	(5.3)	0.655
Satisfactory	40	(17.2)	14	(15.6)	54	(16.8)	
Good	98	(42.2)	45	(50.0)	143	(44.4)	
Very good	81	(34.9)	27	(30.0)	108	(33.5)	
Total	232	(100.0)	90	(100.0)	322	(100.0)	
Chronic disease							
Yes	75	(32.6)	19	(22.6)	94	(29.9)	0.104
No	128	(55.7)	49	(58.3)	177	(56.4)	
Do not know	27	(11.7)	16	(19.0)	43	(13.7)	
Total	230	(100.0)	84	(100.0)	314	(100.0)	
Efficacy of TRM							
Poor	10	(4.3)	5	(5.4)	15	(4.6)	0.952
Satisfactory	24	(10.3)	15	(16.3)	39	(12.0)	
Good	105	(45.3)	30	(32.6)	135	(41.7)	
Very good	93	(40.1)	42	(45.7)	135	(41.7)	
Safe use of TRM							
Poor	10	(4.3)	8	(8.7)	18	(5.6)	0.912
Satisfactory	46	(19.8)	14	(15.2)	60	(18.5)	
Good	115	(49.6)	47	(51.1)	162	(50.0)	
Very good	61	(26.3)	23	(25.0)	84	(25.9)	

**Table 2:** Insurance status by use of traditional medicine.

	Traditional medicine utilisation		Total	*P* value
	TRM users	Non-TRM users		
	*N* (%)	*N* (%)	*N* (%)	
Health insurance status				
Insured	198 (85.3%)	34 (14.7%)	232 (71.6%)	0.527
Uninsured	81 (88.0%)	11 (12.0%)	92 (28.4%)	
Total	279 (86.1%)	45 (13.9%)	324 (100%)	

**Table 3:** Selected survey questions regarding TRM use by health insurance status.

	Health insurance status						*P* value
	Insured		Uninsured		Total		
	*N*	(%)	*N*	(%)	*N*	(%)	
Sources of TRM							
Consult TMP	58	(25.0)	12	(13.0)	70	(21.6)	<0.001
Self-administered	105	(45.3)	50	(54.3)	155	(47.8)	<0.001
Pharmacy shop	48	(20.7)	22	(23.9)	70	(21.6)	0.024
Open markets	21	(9.1)	8	(8.7)	29	(9.0)	0.033
Frequency of TRM use							
None	34	(14.7)	11	(12.0)	45	(13.9)	—
Once	36	(15.5)	22	(23.9)	58	(17.9)	0.066
Two times	62	(26.7)	24	(26.1)	86	(26.5)	<0.001
≥ three times	100	(43.1)	35	(38.0)	135	(41.7)	<0.001
Co-TRM use with orthodox medicine							
Yes	65	(32.8)	21	(26.3)	86	(30.9)	<0.001
No	133	(67.2)	59	(73.8)	192	(69.1)	<0.001
Disclosure of TRM to health care professionals							
Yes	21	(10.6)	13	(16.0)	34	(12.2)	0.170
No	177	(89.4)	68	(84.0)	245	(87.8)	<0.001
Endorsing full integration of TRM into health system							
Yes	210	(90.5)	79	(85.9)	289	(89.2)	0.224
No	22	(9.5)	13	(14.1)	35	(10.8)	

**Table 4:** Factors associated with TRM use among the insured and the uninsured in multiple logistic models.

Factors	Use of TRM by the insured				Use of TRM by the uninsured			
	*B*	OR	95% CI	*P* value	*B*	OR	95% CI	*P* value
Effectiveness of TRM								
Less effective			1.00					
More effective	1.476	4.374	1.753–10.913	0.002^*∗*^	1.219	3.383	0.869–13.170	0.039^*∗*^
Nature of occupation								
Self-employed			1.00					
Public servant	0.437	1.547	0.852–2.811	0.152	−5.687	2.003	0.000–3.592	0.110
Work experience								
≤5 years			1.00					
>5 years	−0.639	1.528	1.309–1.900	0.019^*∗*^	0.966	2.628	0.726–9.513	0.141

1.00 = reference; OR = odds ratio; CI = confidence interval; TRM = traditional medicine; ^*∗*^statistically significant at *P* ≤ 0.05.

## References

[1] Gyasi R. M., Asante F., Segbefia A. Y. (2015). Does spatial location matter? Traditional therapy utilisation among the general population in a Ghanaian rural and urban setting. *Complementary Therapies in Medicine*.

[2] Hughes G. D., Aboyade O. M., Clark B. L., Puoane T. R. (2013). The prevalence of traditional herbal medicine use among hypertensives living in South African communities. *BMC Complementary and Alternative Medicine*.

[3] Hwang J. H., Han D. W., Yoo E. K., Kim W.-Y. (2014). The utilisation of complementary and alternative medicine (CAM) among ethnic minorities in South Korea. *BMC Complementary and Alternative Medicine*.

[4] Kaadaaga H. F., Ajeani J., Ononge S. (2014). Prevalence and factors associated with use of herbal medicine among women attending an infertility clinic in Uganda. *BMC Complementary and Alternative Medicine*.

[5] Eisenberg D. M., Davis R. B., Ettner S. L. (1998). Trends in alternative medicine use in the United States, 1990–1997: results of a follow-up national survey. *Journal of the American Medical Association*.

[6] Pan S.-Y., Litscher G., Gao S.-H. (2014). Historical perspective of traditional indigenous medical practices: the current renaissance and conservation of herbal resources. *Evidence-Based Complementary and Alternative Medicine*.

[7] Tindle H. A., Davis R. B., Phillips R. S., Eisenberg D. M. (2005). Trends in use of complementary and alternative medicine by us adults: 1997–2002. *Alternative Therapies in Health and Medicine*.

[8] WHO (2013). *The WHO Traditional Medicine Strategy 2014–2023*.

[9] Dubey N. K., Kumar R., Tripathi P. (2004). Global promotion of herbal medicine: india's opportunity. *Current Science*.

[10] Sharma A., Shanker C., Tyagi L. K., Singh M., Rao V. (2008). Herbal medicine for market potential in India: an overview. *Academic Journal of Plant Sciences*.

[11] Peltzer K., Mngqundaniso N. (2008). Patients consulting traditional health practitioners in the context of HIV/AIDS in urban areas in KwaZulu-Natal, South Africa. *African Journal of Traditional, Complementary and Alternative*.

[12] Gyasi R. M., Tagoe-Darko E., Mensah C. M. (2013). Use of traditional medicine by HIV/AIDS patients in Kumasi Metropolis, Ghana: a cross-sectional survey. *American International Journal of Contemporary Research*.

[13] Gyasi R. M., Mensah C. M., Siaw L. P. (2015). Predictors of traditional medicines utilisation in the Ghanaian health care practice: interrogating the Ashanti Region. *Journal of Community Health*.

[14] Ghana Statistical Service (2012). *Population and Housing Census, 2010 Summary of Report of Final Results*.

[15] Apt N. A., Maharaj P. (2013). Older people in rural Ghana: health and health seeking behaviours. *Ageing and Health in Africa*.

[16] Gyasi R. M., Mensah C. M., Adjei P. O., Agyemang S. (2011). Public perceptions of the role of traditional medicine in the health care delivery system in ghana. *Global Journal of Health Science*.

[17] World Health Assembly (WHA) (2009). Traditional medicine. *Sixty-Second World Health Assembly, Geneva, 18–22*.

[18] Xu K., Evans D. B., Carrin G., Aguilar-Rivera A. M., Musgrove P., Evans T. (2007). Protecting households from catastrophic health spending. *Health Affairs*.

[19] Gobah F. K., Zhang L. (2011). The national health insurance scheme in ghana: prospects and challenges: a cross-sectional evidence. *Global Journal of Health Science*.

[20] Blanchet N. J., Fink G., Osei-Akoto I. (2012). The effect of Ghana's National Health Insurance Scheme on health care utilisation. *Ghana Medical Journal*.

[21] Osei-Akoto I. (2012). Demand for voluntary health insurance by the poor in developing countries: evidence from rural Ghana. *Working Paper*.

[22] Atim C., Grey S., Apoya P., Anie S. J., Aikins M. (2001). *A Survey of Health Financing Schemes in Ghana*.

[23] Gilson L. (1997). The lessons of user fee experience in Africa. *Health Policy and Planning*.

[24] Palmer N., Mueller D. H., Gilson L., Mills A., Haines A. (2004). Health financing to promote access in low income settings—how much do we know?. *The Lancet*.

[25] Chankova S., Sulzbach S., Diop F. (2008). Impact of mutual health organizations: evidence from West Africa. *Health Policy and Planning*.

[26] Hjortsberg C. (2003). Why do the sick not utilise health care? The case of Zambia. *Health Economics*.

[27] WHO (2002). Promoting rational use of medicines: core components. *WHO Policy Perspectives on Medicines*.

[28] Bodeker G., Kronenberg F. (2002). A public health agenda for traditional, complementary, and alternative medicine. *The American Journal of Public Health*.

[29] Astin J. A. (1998). Why patients use alternative medicine: results of a national study. *Journal of the American Medical Association*.

[30] Eisenberg D. M., Kessler R. C., Foster C., Norlock F. E., Calkins D. R., Delbanco T. L. (1993). Unconventional medicine in the United States: prevalence, costs, and patterns of use. *The New England Journal of Medicine*.

[31] MacLennan A. H., Wilson D. H., Taylor A. W. (1996). Prevalence and cost of alternative medicine in Australia. *The Lancet*.

[32] Chen F.-P., Chen T.-J., Kung Y.-Y. (2007). Use frequency of traditional Chinese medicine in Taiwan. *BMC Health Services Research*.

[33] Carrin G., Doetinchem O., Kirigia J., Mathauer I., Musango L. (2008). Social health insurance: how feasible is its expansion in the African region?. *Development Issues*.

[34] Witter S., Garshong B. (2009). Something old or something new? Social health insurance in Ghana. *BMC International Health and Human Rights*.

[35] Bennett S., Gamble-Kelley A., Silvers B. (2004). *21 Questions on CBHF: An Overview of Community-Based Health Financing*.

[36] Barimah K. B. (2013). Traditional healers as service providers in Ghana's National Health Insurance Scheme: the wrong way forward?. *Global Public Health*.

[37] United Nations Development Programme (UNDP) (2007). *Towards a More Inclusive Society. Ghana Human Development Report 2007*.

[38] Lwanga S., Lemeshow S. (1991). *Sample Size Determination in Health Studies: A Practical Manual*.

[39] Fisher A. A., Laing E. J., Stoeckel E. J., Townsend W. J. (1998). *Handbook for Family Planning Operations Research Design*.

[40] Elolemy A. T., Albedah A. M. N. (2012). Public knowledge, attitude and practice of complementary and alternative medicine in Riyadh region, Saudi Arabia. *Oman Medical Journal*.

[41] Israel M., Hay I. (2006). *Research Ethics for Social Scientists: Between Ethical Conduct and Regulatory Compliance*.

[42] World Medical Association Declaration of Helsinki.

[43] Ness J., Cirillo D. J., Weir D. R., Nisly N. L., Wallace R. B. (2005). Use of complementary medicine in older Americans: results from the health and retirement study. *Gerontologist*.

[44] Gyasi R. M., Siaw L. P., Mensah C. M. (2015). Prevalence and pattern of traditional medical therapy utilisation in Kumasi metropolis and Sekyere south district, Ghana. *Journal of Ethnopharmacology*.

[45] Onyiapat J.-L. E., Okoronkwo I. L., Ogbonnaya N. P. (2011). Complementary and alternative medicine use among adults in Enugu, Nigeria. *BMC Complementary and Alternative Medicine*.

[46] Kav T. (2009). Use of complementary and alternative medicine: a survey in Turkish gastroenterology patients. *BMC Complementary and Alternative Medicine*.

[47] Eddouks M., Maghrani M., Lemhadri A., Ouahidi M.-L., Jouad H. (2002). Ethnopharmacological survey of medicinal plants used for the treatment of diabetes mellitus, hypertension and cardiac diseases in the south-east region of Morocco (Tafilalet). *Journal of Ethnopharmacology*.

[48] Appelbaum Belisle H., Hennink M., Ordóñez C. E. (2015). Concurrent use of traditional medicine and ART: perspectives of patients, providers and traditional healers in Durban, South Africa. *Global Public Health*.

[49] Gyasi R. M. (2014). *Analysis of factors influencing traditional medicines utilization in Ghana: evidence from Ashanti Region [M.S. thesis]*.

[50] Sato A., Costa-i- Font J. (2012). Does culture matter at all in explaining why people still use traditional medicine?. *Working Paper*.

[51] Kretchy I. A., Owusu-Daaku F. T., Danquah S. A. (2014). Mental health in hypertension: assessing symptoms of anxiety, depression and stress on anti-hypertensive medication adherence. *International Journal of Mental Health Systems*.

[52] Buor D. (2008). Analysing the socio-spatial inequities in the access of health services in sub-Saharan Africa: interrogating geographical imbalances in the uptake of health care. *Professorial Inaugural Lecture*.

[53] Gaffney L., Smith C. (2004). Complementary and alternative medicine in obstetrics. *Birth Issues*.

[54] Van den Boom G. J. M., Nsowah-Nuamah N. N. N., Overbosch G. B. (2004). Health care provision and self-medication in Ghana. *JEL Classification*.

